# Association Between Abnormal DNA Methylation and Altered Transcriptome in Muscle Five Years After Critical Illness

**DOI:** 10.1002/jcsm.70170

**Published:** 2026-01-19

**Authors:** Ceren Uzun Ayar, Fabian Güiza, Inge Derese, Greet Van den Berghe, Ilse Vanhorebeek

**Affiliations:** ^1^ Laboratory of Intensive Care Medicine, Department of Cellular and Molecular Medicine KU Leuven Leuven Belgium; ^2^ Clinical Division of Intensive Care Medicine University Hospitals Leuven Leuven Belgium

**Keywords:** critical illness, DNA methylation, epigenetics, muscle weakness, post‐intensive care syndrome, transcriptome

## Abstract

**Background:**

Critically ill patients requiring intensive care unit (ICU) admission suffer from muscle weakness that persists for years. Recently, altered RNA expression was documented in muscle of former ICU patients 5 years after critical illness that suggested disrupted mitochondrial function, disturbed lipid metabolism and fibrosis, of which many associated with the former patients' long‐term loss of muscle strength. We hypothesized that abnormal DNA methylation detectable years after critical illness associates with these abnormal RNA expression patterns, as a potential biological basis for the persistent loss of muscle strength.

**Methods:**

Genome‐wide DNA methylation was assessed (Infiniumv2‐HumanMethylationEPIC‐BeadChips) in skeletal muscle biopsies from 118 former ICU patients harvested 5 years after critical illness (79.6% male, median 58 years, median BMI 27.3 kg/m^2^) and 30 controls who never required ICU admission (76.7% male, median 61 years, median BMI 26.4 kg/m^2^). Differentially methylated positions (DMPs) in former patients versus controls were identified, adjusting for age, sex, and BMI (minfi‐package in R, Benjamini–Hochberg false‐discovery‐rate < 0.05), followed by pathway over‐representation of affected genes. Spearman correlations between DMP methylation and RNA expression were compared among groups of RNA with Z‐test and Kolmogorov–Smirnov test. Risk factors for abnormal DNA methylation were identified with multivariable linear regression.

**Results:**

As compared with controls, former ICU patients showed 7379 DMPs (average difference 2.6% ranging up to 24.9%). They were associated with 1334 unique genes, enriched for muscle contraction, vascular development, cell differentiation and signal transduction. DMPs correlated more strongly with differentially expressed RNAs (DERNAs) than with non‐differentially expressed RNAs (18.1% vs. 1.7% correlations with |rho| > 0.3, *p* < 2.2 × 10^−16^). Such correlations were more abundant among DERNAs associated with reduced muscle strength vs. those not associated (24.4% vs. 12.5%), also within the previously identified disrupted pathways (mitochondrial function 23.3% vs. 10.9%, lipid metabolism 15.9% vs. 7.2%, fibrosis 44.3% vs. 5.8%, all *p* < 2.2 × 10^−16^). Older age, female sex, in‐ICU treatment with glucocorticoids, benzodiazepines, early parenteral nutrition and opioids and insulin and antipsychotic medication at follow‐up were most notably associated with more abnormal DNA methylation.

**Conclusions:**

Abnormal DNA methylation in muscle biopsied 5 years after critical illness associated with long‐term altered RNA expression that has been linked to lower muscle strength. These data suggest a possible epigenetic basis for this long‐term sequel after critical illness. Abnormal DNA methylation was also found to associate with (possibly) avoidable risk factors during and after ICU stay. These findings may open perspectives for prevention and possibly treatment of long‐term muscle weakness after critical illness.

## 1 Introduction

Critically ill patients requiring intensive care often develop multiple organ failure. Many are confronted with intensive care unit‐acquired weakness (ICUAW), which affects both limb and respiratory muscles and has been associated with worse outcomes [1]. Although advances in intensive care have improved survival, many ICU survivors continue to experience long‐term complications [2, 3]. Among these complications, persistent muscle wasting and weakness may be particularly prominent and can severely impact quality of life [4, 5].

Ample mechanistic research on muscle weakness during critical illness generated substantial pathophysiological insights [1, 6]. However, data on the molecular and structural features underlying persistent weakness in ICU survivors are scarce. A small study (n=10) suggested normalization of muscle proteolysis, autophagy, inflammation, and mitochondrial structure within 6 months post‐ICU, whereas the persistent weakness was associated with impaired regeneration and fibrosis [7, 8]. Our recent large transcriptome study on muscle of 115 former ICU patients 5 years after critical illness revealed abnormal RNA expression profiles as compared with controls, pointing to impaired mitochondrial function, disturbed lipid metabolism and enhanced fibrosis, associated with lower long‐term muscle strength [9]. Some alterations were partly attributable to potentially avoidable risk factors such as in‐ICU early parenteral nutrition and glucocorticoid treatment. While these findings provide valuable insights, they do not fully explain the molecular mechanisms underlying the persistent transcriptional and functional changes.

A plausible mechanism linking transient adverse exposures to long‐term transcriptional alterations and functional impairments could be the development of abnormal epigenetic marks, particularly altered DNA methylation [10]. Skeletal muscle is susceptible to changes in DNA methylation that regulate gene programs involved in muscle development and regeneration, metabolism and muscle‐specific functions [11, 12]. Notably, muscle from critically ill patients on ICU Day 8 showed a DNA methylation profile distinct from controls, affecting genes highly relevant for muscle structure and function [13]. If such changes persist, serving as a molecular memory of critical illness, they could affect gene expression long after ICU discharge.

**FIGURE 1:**
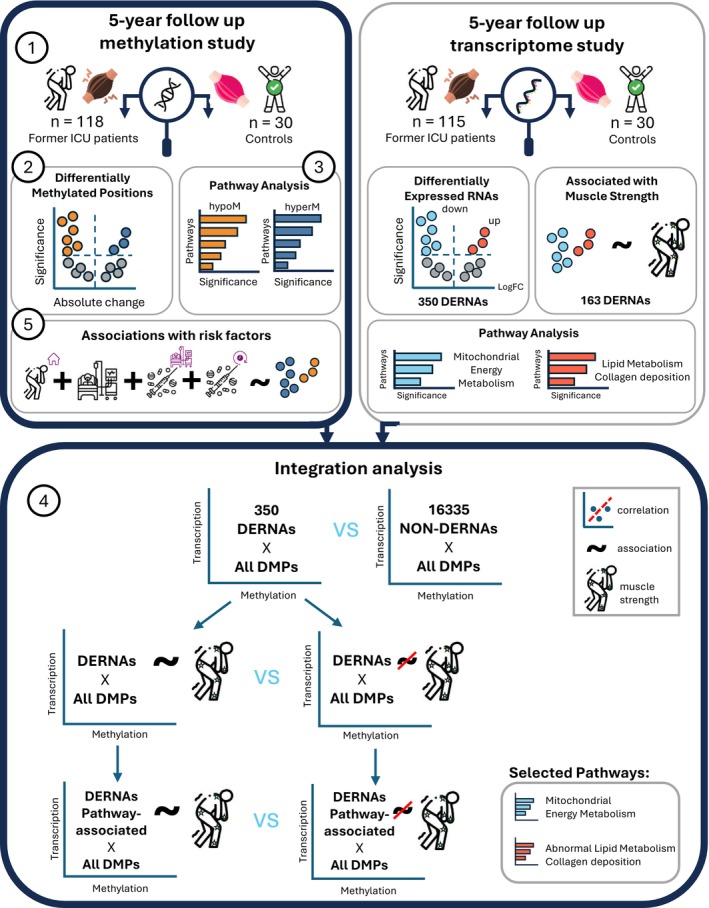
Study design. Step 1: A genome‐wide DNA methylation analysis was performed on muscle of former ICU patients, who were studied 5 years after critical illness, and on muscle of controls with no ICU history. Step 2: DNA positions differentially methylated in former ICU patients as compared with controls were identified, adjusting for age, sex, and BMI. Step 3: Differentially methylated genes were annotated, and enriched pathways were identified using GOBP, KEGG and Reactome databases. Step 4: Beta values (methylation level) of DMPs were correlated with normalized RNA expression data from our previous transcriptome study of the same cohort [9], which had identified 350 differentially expressed RNAs (DERNAs) that were enriched in mitochondrial, lipid, and fibrosis‐related pathways and associated with decreased long‐term muscle strength. Percentages of correlations with 1|rho| > 0.3 and overall distributions of rho values were compared across RNA groups (DERNAs vs. non‐DERNAs, strength‐associated vs. non–strength‐associated, and pathway‐specific subsets). Step 5: Risk factors associated with abnormal DNA methylation in former ICU patients were identified. Abbreviations: DMP, differentially methylated position; DERNA, differentially expressed RNA; GOBP, Gene Ontology biological process; HypoM, hypomethylated; HyperM, hypermethylated; ICU, intensive care unit.

We hypothesized that former ICU patients exhibit long‐term aberrant muscle DNA methylation profiles, which may associate with the persistent abnormal RNA expression. To test this hypothesis, we compared muscle DNA methylation profiles of former ICU patients 5 years post‐ICU with those of controls who never required an ICU admission, identified affected pathways, and assessed whether any DNA methylation alterations correlate with RNA expression. In addition, we identified (modifiable) risk factors for long‐term abnormal DNA methylation (Figure 1).

## 2 Methods

### 2.1 Study Design and Participants

This is a pre‐planned prospective secondary analysis of the multicentre EPaNIC trial (ClinicalTrials.gov‐NCT00512122) and its 5‐year follow‐up [5, 14]. This trial included 4640 adult critically ill patients to study the impact of early supplementation of insufficient enteral nutrition with parenteral nutrition (early‐PN) as compared with omitting supplemental parenteral nutrition in the first week in ICU (late‐PN). All patients received enteral nutrition as soon as possible, parenteral trace elements, minerals and vitamins, insulin infusions to maintain normoglycaemia, and physiotherapy according to standardized rehabilitation protocols [14, 15].

**FIGURE 2:**
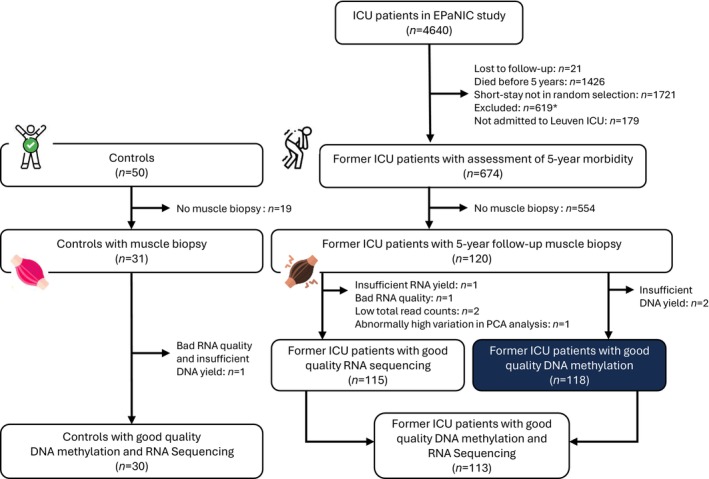
Flowchart of study participants. Of the 4640 patients who had been enrolled in the EPaNIC study, 674 participated in a follow‐up assessment 5 years later, in comparison with 50 control individuals with comparable age, sex and BMI as the former EPaNIC patients. The controls had never required an ICU admission, did not suffer from conditions that could confound the morbidity endpoints and were recruited via primary care givers and outpatient clinics. At that 5‐year follow‐up time point, 120 former ICU patients and 31 of the controls gave a muscle biopsy. DNA yield was insufficient for two patients and one control. DNA methylation analyses of all remaining samples passed all quality controls, thus good quality DNA methylation data were available for analysis for 118 patients and 30 controls. *Patients with pre‐ICU neuromuscular disorders, unable to walk without assistance prior to ICU or other disabilities present before follow‐up potentially confounding morbidity endpoints, refusing participation or not contactable were excluded. Abbreviation: ICU, intensive care unit.

Long‐term physical morbidity of 674 EPaNIC patients was investigated 5 years after ICU admission (Figure 2) [5]. This cohort resulted from all long‐stay patients (ICU stay ≥ 8 days as 75th percentile of ICU length of stay) being eligible together with a random, computer‐generated subset of short‐stay patients with a similar distribution of admission diagnoses. Patients suffering from conditions that could confound the morbidity endpoints were a priori excluded. Such conditions comprised pre‐existing neuromuscular disorders or inability to walk without assistance before ICU admission, or other physical disabilities (cardiac assist device, pulmonary resection, psychiatric disease, dementia, vegetative state, in hospital/rehabilitation centre/nursing home). In parallel, 50 individuals with comparable age, sex and BMI who had never required an ICU admission and did not suffer from conditions that could confound the morbidity endpoints were recruited via primary care givers and outpatient clinics (Figure 2). For this study, we selected all participants who allowed a muscle biopsy collection, including 120 former ICU patients and 31 controls (Figure 2). In vivo needle biopsies were taken percutaneously from the musculus vastus lateralis of the quadriceps femoris at mid‐thigh level (Bergström technique), after local anaesthesia with lidocaine 2%. Biopsies were snap‐frozen in liquid nitrogen and stored at −80°C for molecular analyses. Analyses were performed blinded for patient‐control status.

The study protocol and informed consent forms were approved by the Leuven University Hospital Ethics Committee (ML4190). All patients or their next‐of‐kin provided written informed consent for participation in the EPaNIC study. All former ICU patients and controls provided written informed consent for participation in the follow‐up study.

### 2.2 DNA Extraction and Differential DNA Methylation Analysis

Genomic DNA was extracted using proteinase K tissue digestion and isopropanol DNA precipitation. DNA concentrations were quantified with the Qubit 3.0 fluorometer (Thermo Fisher Scientific, Waltham, MA). DNA yield was insufficient for two former ICU patients and one control (Figure 2). DNA samples were shipped to Life&Brain GmbH (Bonn, Germany) where DNA was bisulphite converted (EZ‐96 DNA Methylation‐Lightning Kit (Deep‐Well), Zymo Research, Irvine, CA) and DNA methylation analyses were performed with the use of Infinium’s HumanMethylationEPIC v2.0 BeadChips (Illumina Inc., San Diego, CA) that interrogate more than 935 000 CpG sites. Patient and control samples were distributed over 25 chips. Sample quality assessments were performed using detection *p* values, q‐plot, signal intensities, density plots and principal component analysis. Raw intensities were normalized with stratified quantile normalization. Probes with mean detection *p >* 0.01 in at least 50% of samples (indicating poor detection of signal above noise) and probes spanning known single nucleotide polymorphisms were excluded. Differentially methylated positions (DMPs) were identified on M‐values (log_2_ ratios of the intensities of methylated probes versus unmethylated probes), adjusting for sex, age and BMI. The analyses were conducted using the minfi package (v1.46.0) in R [16]. Multiple testing correction was applied using the Benjamini–Hochberg procedure, with false‐discovery‐rate (FDR) < 0.05. Gene annotations, including associated genes, and gene regions were extracted from the Illumina EPIC annotation file (IlluminaHumanMethylationEPICv2anno.20a1.hg38, v1.0). Gene regions were categorized as promoter region (where transcription is initiated), 5′ untranslated region (5′UTR, involved in translation regulation), gene body (coding region), 3′UTR (a post‐transcriptional regulatory region) and intergenic region (not linked to any known transcript).

### 2.3 Pathway Enrichment Analysis of Differentially Methylated Genes

We identified all differentially methylated genes (DMGs) as those harbouring at least one DMP. To assess the biological significance of these DMGs, pathway overrepresentation analysis was performed using EnrichR (v1.40.2) [17], evaluating enrichment in the Gene Ontology Biological Process (GOBP) and Reactome databases. In addition, we performed pathway enrichment analysis at the level of DMPs with the R package missMethyl (v1.40.3) [18], using all evaluated DNA positions as the background and with use of the GOBP and Kyoto Encyclopedia of Genes and Genomes (KEGG) databases. This procedure accounts for the varying number of DMPs per gene, thereby reducing bias in overrepresentation analyses. Both analyses were performed separately for hypermethylated and hypomethylated DMPs, and all DMPs combined, with adjustment of *p* values for multiple testing using the Benjamini–Hochberg procedure (FDR < 0.05).

### 2.4 Differential RNA Expression Analysis

We previously performed a transcriptome analysis on RNA extracted from the same set of muscle biopsies collected during the EPaNIC 5‐year follow‐up study, retaining good quality data for 115 former ICU patients and 30 controls (Figures 1 and 2, Method S1) [9]. In brief, RNA sequencing had been performed by the UZ/KU Leuven Genomics Core Facility and differential expression analysis was performed with the DESeq2 package (v1.46.0) in R, adjusting for age, sex and BMI, and applying the Benjamini–Hochberg procedure for multiple testing correction (FDR < 0.05). In that study, we have identified 350 differentially expressed RNAs (DERNAs) in ICU patients 5 year after ICU admission as compared with controls. Pathway analysis (including over‐representation and gene set enrichment) revealed that down‐regulated RNAs showed enrichment in mitochondrial ATP synthesis and cellular respiration pathways, while up‐regulated RNAs were enriched in AMPK and PPAR signalling (involved in lipid metabolism) as well as collagen formation pathways. The expression of 163 of these 350 RNAs showed a significant association with the former ICU patients' long‐term reduced muscle strength [9].

### 2.5 Correlation of Differential DNA Methylation With RNA Expression

To investigate whether differential DNA methylation associates with differential RNA expression in former ICU patients 5 years after ICU admission, we used the stats package in R (v4.42) to perform Spearman correlation analyses between methylation beta‐values (indicating degree of methylation ranging from 0 as 0% methylation to 1 as 100% methylation) and normalized RNA expression data. DNA methylation can regulate gene expression both in cis and in trans, including effects on genes located on different chromosomes [19]. This is enabled by the 3D genome architecture, where chromatin looping and inter‐chromosomal contacts bring distant loci into proximity [20]. To also capture these long‐range effects, we performed these analyses with all DMPs. This unbiased approach allows detection of regulatory associations beyond linear proximity.

First, we correlated all DMPs with all differentially expressed RNAs (DERNAs) on one hand and, for comparison, with all non‐differentially expressed RNAs (non‐DERNAs) on the other hand. Then, we focused separately on the correlations within the subset of DERNAs for which we previously found an association with long‐term muscle strength and within the DERNAs not associated with muscle strength. Next, to explore pathway‐specific relationships, we zoomed in on the correlations of the DMPs with the DERNAs involved in each of the previously identified persistently disturbed pathways (mitochondrial energy metabolism, lipid metabolism and fibrosis), separately for DERNAs associated and those not associated with muscle strength. Each time we calculated the percentage of correlations with |rho| > 0.3, which were considered biologically relevant, and compared them with two‐proportion Z‐tests. For the same groups of RNAs, we also compared the resulting rho value distributions by calculating empirical cumulative distribution functions (ECDFs). ECDFs represent the proportion of observations less than or equal to each correlation coefficient (rho), providing a stepwise, nonparametric view of each RNA group's distribution. Each curve rises from 0 to 1, capturing the cumulative distribution of correlation values within a group. Differences between groups were assessed using the asymptotic two‐sample Kolmogorov–Smirnov (KS) test, where the maximum vertical distance between two curves is quantified by the test statistic D. A larger D value indicates a greater difference between the two distributions.

### 2.6 Identification of Risk Factors Associated With Abnormal DNA Methylation in Former ICU Patients

To identify risk factors that are independently associated with the documented abnormal long‐term DNA methylation levels, we constructed a multivariable linear regression model (limma v3.62.2 [21] in R) for each of the identified DMPs, with the corresponding beta‐value as dependent variable and the potential risk factors as independent variables. Considered risk factors included demographics (sex, age and BMI at 5‐year follow‐up), ICU baseline characteristics (reason for ICU admission, APACHE‐II score, sepsis upon admission, diabetes, malignancy), in‐ICU complications and treatments (ICU length of stay, duration of mechanical ventilation, duration of hemodynamic support, early‐PN versus late‐PN, duration of treatment with hypnotics, alpha2 agonists, glucocorticoids, benzodiazepines or opioids, and acquisition of a new infection in ICU) and medications taken at the time of follow‐up (insulin, glucocorticoids, betablockers, antidepressants, antipsychotics, oral antidiabetics, statins). Associations with a *p* < 0.05 were considered statistically significant and were visualized with the ggplot2 (v3.5.1) package [22] in R.

### 2.7 Statistical Analyses

Participants' characteristics and measures of muscle strength are presented as median and interquartile range (IQR) or number and percentage, as indicated. Former ICU patients and controls were univariably compared for these data with chi‐square tests for proportions and with Wilcoxon signed rank tests for continuous data, or adjusted for covariates (age, sex and BMI) with multivariable linear regression analyses (for muscle strength). All statistical analyses were performed with R (v4.4.2). Sample size was determined by available muscle biopsies. Statistical power was not a priori estimated, as anticipated effect sizes were unclear but deemed sufficiently large based on relevant findings in other genome‐wide DNA methylation studies with smaller or similar sample sizes [13, 23].

## 3 Results

### 3.1 Participants

Sufficient DNA was available from 118 patients and 30 healthy controls (Figure 2). All the samples passed the quality controls, and no outliers or batch effects were observed (Figures S1–S5). Hence, all samples were included in downstream analyses. Baseline characteristics of the former patients and controls are reported in Table 1.

**TABLE 1:** Participant characteristics and long‐term muscle strength.

Characteristic	Controls *n* = 30	Former ICU patients *n* = 118	*p*
Demographics at 5‐year follow‐up			
Age (years), median (IQR)	61 (57–65)	58 (50–66)	
Male sex, *n* (%)	23 (76.7)	94 (79.6)	
BMI (kg/m^2^), median (IQR)	26.4 (23.6–28.1)	27.3 (23.8–30.4)	
White race, *n* (%)	30 (100.0)	118 (100.0)	
Baseline factors at ICU‐admission			
History of diabetes, *n* (%)	2 (6.7)	10 (8.5)	
History of malignancy, *n* (%)	3 (10.0)	14 (11.9)	
Pre‐ICU‐admission dialysis, *n* (%)	0 (0.0)	0 (0.0)	
APACHE‐II score first 24 h, median (IQR)		26 (15–32)	
Admission diagnosis, *n* (%)			
Cardiac surgery		43 (36.4)	
Elective other surgery		11 (9.3)	
Emergency other surgery		58 (49.2)	
Medical diagnosis		6 (5.1)	
Sepsis upon ICU‐admission		28 (23.7)	
Complications and treatments in ICU			
ICU‐stay (days), median (IQR)		5 (2–14)	
ICU‐stay of at least 8 days, *n* (%)		44 (37.3)	
Mechanical ventilation (days), median (IQR)		3 (1–9)	
Hemodynamic support (days), median (IQR)		2 (1–4)	
Early‐PN, *n* (%)		61 (51.7)	
Antibiotics (days), median (IQR)		2 (1–11)	
New infection, *n* (%)		36 (30.5)	
Hypoglycaemia (days), *n* (%)		0 (0–0)	
Glucocorticoids, *n* (%)		34 (28.8)	
Glucocorticoids (days), median (IQR)		0 (0–1)	
Opioids (days), median (IQR)		3 (2–11)	
Benzodiazepines (days), median (IQR)		2 (0–9)	
Alpha2‐agonist (days), median (IQR)		0 (0–0)	
Hypnotics (days), median (IQR)		2 (1–4)	
Current medication at 5‐year follow‐up			
Insulin therapy, *n* (%)	0 (0.0)	7 (5.9)	
Oral anti‐diabetic medication, *n* (%)	0 (0.0)	13 (11.0)	
Glucocorticoids, *n* (%)	0 (0.0)	8 (6.8)	
Beta‐blockers, *n* (%)	0 (0.0)	53 (44.9)	
Anti‐depressants, *n* (%)	0 (0.0)	15 (12.7)	
Antipsychotics, *n* (%)	0 (0.0)	3 (2.5)	
Statins, *n* (%)	0 (0.0)	54 (45.8)	
Muscle strength at 5‐year follow‐up^a^			
HGS dominant hand (% pred), median (IQR)	104 (92–120)	93 (80–107)	0.0024
HGS non‐dominant hand (% pred), median (IQR)	114 (102–119)	104 (89–116)	0.10
Strength shoulder (%pred), median (IQR)	98 (86–112)	93 (81–107)	0.032
Strength elbow (%pred), median (IQR)	99 (88–115)	85 (75–100)	0.0001
Strength wrist (%pred), median (IQR)	109 (97–123)	98 (87–115)	0.040
Strength hip (%pred), median (IQR)	164 (136–180)	141 (122–158)	0.015
Strength knee (%pred), median (IQR)	66 (59–74)	53 (45–63)	0.0001
Strength ankle (%pred), median (IQR)	92 (77–111)	71 (61–86)	< 0.0001

Abbreviations: %pred, percentage of predicted; APACHE‐II, acute physiology and chronic health evaluation‐II; BMI, body‐mass index; HGS, handgrip‐strength; ICU, intensive care unit; IQR, interquartile range; PN, parenteral nutrition.

^a^ Denotes *p* values adjusted for age, sex and BMI in multivariable linear regression analysis.

### 3.2 Differential DNA Methylation in Former ICU Patients as Compared With Controls

**FIGURE 3:**
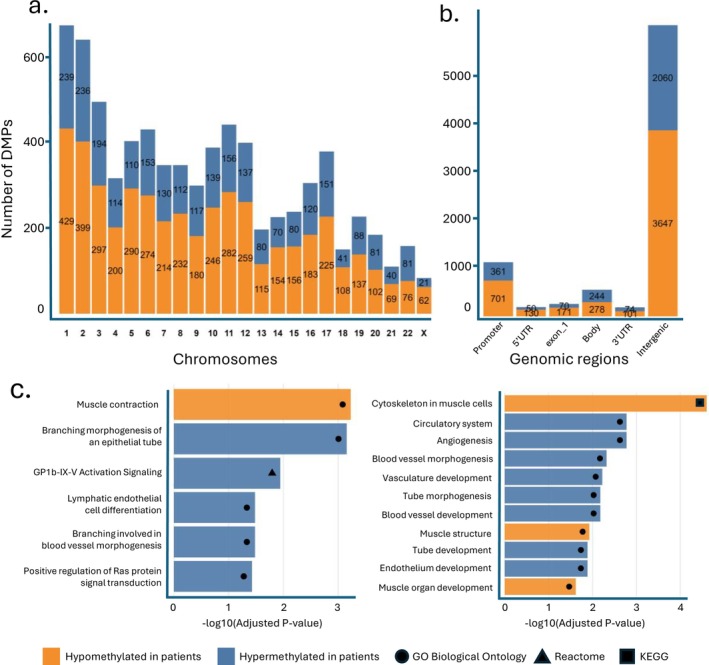
Location of differentially methylated positions and enrichment analysis of affected pathways. (a) Distribution of the differentially methylated positions across the chromosomes. (b) Genomic region in relation to known gene transcripts for the 7379 CpG sites that were differentially methylated in former critically ill patients as compared with controls. Gene regions are categorized as promoter, 5′UTR, gene body, 3′UTR and intergenic region. The height of the bars indicates the total number of CpG sites with orange and blue representing hypo‐ and hypermethylation, respectively. CpG sites overlapping multiple transcript regions are counted in each relevant genomic region. (c) Pathway enrichment analysis of hypermethylated and hypomethylated DMPs. Enrichment was assessed with EnrichR (GOBP, Reactome) in the left panel and with missMethyl (GOBP, KEGG) in the right panel (FDR < 0.05). Detailed pathway results are provided in Tables S3–S4. Abbreviations: ICU, intensive care unit; UTR, untranslated region; DMP, differentially methylated position; DMG, differentially methylated gene; GOBP, Gene Ontology Biological Process; KEGG, Kyoto Encyclopedia of Genes and Genomes.

After pre‐processing and quality controls, 915 189 of 936 900 CpG sites remained for differential DNA methylation analyses. A total of 7379 CpG sites were differentially methylated in the former ICU patients as compared with the controls (Table S1). Among them, 4689 (63.6%) were hypomethylated and 2690 (36.4%) were hypermethylated in the former ICU patients, with an average effect size of an absolute 2.6% difference in methylation ranging up to 24.8%. The 7379 DMPs were distributed across all autosomes and the X chromosome (Figure 3a). Of the DMPs, 1061 (14.4%) were located within gene promoters, 522 (7.1%) within gene bodies, 180 (2.4%) within the 5′ untranslated region (UTR), 175 (2.4%) within the 3′ UTR, and 5707 (77.3%) were located in intergenic regions (Figure 3b).

### 3.3 Differentially Methylated Genes in Former ICU Patients as Compared With Controls

The DMPs were associated with 1334 unique genes (Table S2). Pathway over‐representation analysis starting from all these DMGs revealed enrichment for the GO terms muscle contraction (GO:0006936) and kinase signalling (GO:0007178) and Reactome terms related to dysregulated coagulation/platelet function (Table S3). Of the 1334 DMGs, 1057 (79.1%) contained a single DMP and 277 (20.9%) contained multiple DMPs. Focusing on the direction of the differential methylation, 779 DMGs contained only hypomethylated DMPs and 534 DMGs contained only hypermethylated DMPs, whereas 21 DMGs contained both hypermethylated and hypomethylated DMPs. Hypomethylated DMGs were enriched for muscle contraction (GO:0006936) (Figure 2c, Table S3). Hypermethylated DMGs were enriched for epithelial and vascular branching morphogenesis (GO:0048754, GO:0001569), lymphatic endothelial cell differentiation (GO:0060836) and Ras protein signal transduction (GO:0046579), and Reactome term related to platelet function (Figure 2c, Table S3). Pathway over‐representation analysis starting from DMPs similarly revealed muscle‐related GO and KEGG terms for hypomethylated DMPs and GO terms related to blood vessel and epithelial tube development for hypermethylated DMPs (Figure 2c, Table S4).

When we compared the 1334 DMGs with the previously identified 350 DERNAs [9], we found 23 common RNAs, of which 22 were protein‐coding and 1 was non‐coding RNA (Table 2). Together, the 23 common RNAs contained 43 DMPs. Of the 16 genes with down‐regulated expression, 11 together contained 17 hypermethylated DMPs, mostly located within the 5′UTR/promoter/exon 1 region; four together contained five hypomethylated DMPs within the 5′UTR/promoter/exon 1 region; and the remaining one contained six hypermethylated DMPs mostly located within the 5′UTR/promoter/exon 1 region and two hypomethylated DMPs within the gene body/3′UTR. Of the seven genes with up‐regulated expression, six together contained 12 hypomethylated DMPs, mostly located within the 5′UTR/promoter/exon 1 region, whereas the other one was hypermethylated at one position in the gene body/3′UTR region.

**TABLE 2:** Common genes identified with DNA methylome and transcriptome studies.

Gene	Gene expression (former ICU patients vs. controls)	Associated with decreased muscle strength	Gene function	CpG site	Methylation status (former ICU patients vs. controls)	Gene region	*P*	*P*_adj_	ΔM	Effect size (%)
ADGRF5	DOWN	Yes	Transmembrane signalling receptor activity	cg00402074	Hyper	TSS200	0.00034	0.047	0.19	6.710
AQP1	DOWN	No	Control of water transport	cg00516678	Hyper	TSS1500	0.00033	0.047	0.18	6.280
ATP6V1FNB	UP	No	Assembly, or the regulation of the V‐ATPase complex	cg27070965	Hypo	TSS1500	0.00037	0.049	−0.15	5.143
DECR1	DOWN	No	Oxidoreductase activity, NADPH binding	cg27040700	Hyper	5UTR; exon_2	0.00013	0.034	0.23	7.896
DIPK2B	DOWN	No	Cellular secretory pathway	cg22010317	Hyper	TSS1500	0.00040	0.050	0.17	5.937
				cg26983645	Hyper	TSS1500	0.00039	0.049	0.19	6.404
DLC1	DOWN	No	GTPase activator activity and protein and lipid binding	cg27378814	Hyper	3UTR; exon_13; exon_14; exon_17; exon_18	0.00000	0.013	0.25	8.491
ENPP5	DOWN	No	NAD + diphosphatase activity and zinc ion binding	cg01609559	Hypo	TSS1500	0.00025	0.042	−0.20	6.870
				cg09539996	Hypo	TSS1500	0.00014	0.034	−0.26	9.012
IDH2	DOWN	No	Oxidoreductase activity, isocitrate dehydrogenase (NADP+) activity, magnesium ion binding	cg00124684	Hypo	TSS200	0.00031	0.046	−0.22	7.595
IQSEC1	DOWN	No	Protein and lipid binding	cg02957897	Hyper	exon_5; exon_7	0.00003	0.023	0.27	9.458
LNX1	DOWN	No	Ubiquitin‐protein transferase activity	cg06495586	Hyper	TSS1500	0.00035	0.047	0.16	5.693
				cg20589120	Hyper	TSS1500	0.00011	0.031	0.15	5.288
MAP 3K7CL	UP	No	Kinase signalling, regulation of apoptosis, cell growth, differentiation	cg02416873	Hypo	TSS1500	0.00001	0.017	−0.24	8.441
				cg14417798	Hypo	5UTR; exon_5; exon_6; exon_7	0.00005	0.026	−0.28	9.797
				cg15251010	Hypo	TSS200; TSS1500	0.00003	0.023	−0.31	10.810
				cg15433195	Hypo	TSS1500	0.00006	0.027	−0.25	8.722
				cg16999170	Hypo	TSS200; TSS1500	0.00036	0.048	−0.26	9.154
METTL7A	UP	No	Bone regeneration and cancer	cg16424082	Hypo	exon_2	0.00027	0.043	−0.25	8.509
NAP1L1	UP	Yes	Chromatin formation and transcriptional regulation	cg10068243	Hyper	3UTR; exon_15	0.00026	0.043	0.17	5.744
NDUFC1	DOWN	No	NADH dehydrogenase (ubiquinone) activity	cg12500690	Hypo	5UTR; exon_1	0.00005	0.025	−0.31	10.698
NNMT	UP	Yes	Methyl‐transferase activity	cg02991558	Hypo	TSS200	0.00029	0.044	−0.19	6.551
				cg21664672	Hypo	TSS200	0.00018	0.037	−0.16	5.627
PTPRB	DOWN	No	Phosphoprotein phosphatase activity	cg19344708	Hyper	exon_2; exon_4	0.00017	0.037	0.13	4.625
SETD9	DOWN	No	Methyl‐transferase activity	cg07905924	Hyper	TSS1500	0.00004	0.023	0.24	8.381
SH3TC2	DOWN	Yes	Myelination and maintenance of nodes of Ranvier	cg03016867	Hyper	TSS1500	0.00004	0.024	0.19	6.535
				cg08688681	Hypo	3UTR; exon_17	0.00003	0.022	−0.27	9.198
				cg08688746	Hypo	3UTR; exon_17	0.00034	0.047	−0.27	9.357
				cg14818279	Hyper	exon_1	0.00030	0.045	0.20	6.845
				cg15502698	Hyper	TSS1500	0.00001	0.017	0.19	6.745
				cg19962468	Hyper	TSS200	0.00026	0.043	0.17	5.987
				cg24815728	Hyper	exon_6	0.00001	0.018	0.26	9.028
				cg26474043	Hyper	TSS1500	0.00005	0.025	0.20	6.751
SH3TC2‐DT	DOWN	Yes	Myelination, stemness, development of neuro‐degenerative diseases	cg03016867	Hyper	exon_1	0.00004	0.024	0.19	6.535
				cg14818279	Hyper	TSS1500	0.00030	0.045	0.20	6.845
				cg15502698	Hyper	exon_1	0.00001	0.017	0.19	6.745
				cg19962468	Hyper	TSS200	0.00026	0.043	0.17	5.987
				cg26474043	Hyper	TSS200	0.00005	0.025	0.20	6.751
SPATA13	DOWN	No	Cell migration	cg27026786	Hyper	exon_2; exon_4	0.00007	0.028	0.23	7.831
SPTB	UP	Yes	Stability of erythrocyte membranes	cg01642461	Hypo	exon_1; exon_2	0.00012	0.032	−0.20	6.921
SYNPO2	UP	Yes	Actin bundling activity and cell migration	cg09757859	Hypo	5UTR; exon_1; exon_3	0.00012	0.033	−0.23	7.959
				cg13541713	Hypo	TSS1500	0.00002	0.021	−0.28	9.631
TRMT9B	DOWN	No	Regulate synaptic growth	cg11209481	Hypo	TSS200	0.00011	0.032	−0.18	6.239

Abbreviations: DM, difference in methylation M‐value; Hyper, hypermethylation; Hypo, hypomethylation; ICU, intensive care unit; *P*_adj_, *p* value adjusted for multiple comparisons; TSS, transcription start site; UTR, untranslated region.

### 3.4 Association of Abnormal DNA Methylation With Abnormal RNA Expression in Former ICU Patients and Controls

**FIGURE 4:**
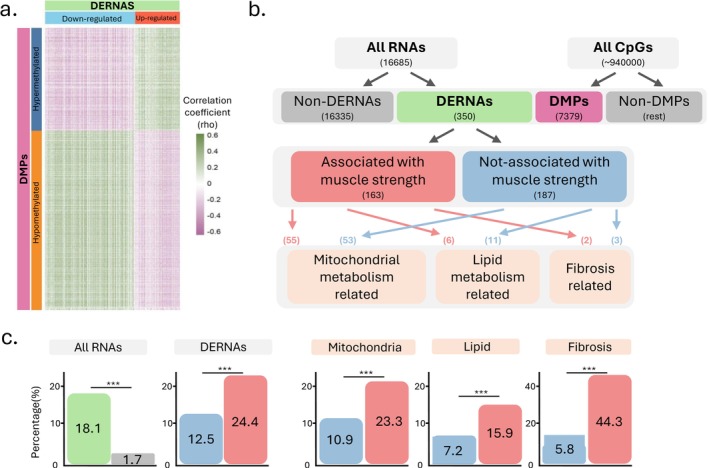
Correlation of differential DNA methylation with RNA expression. (a) Heatmap of Spearman correlations between the beta‐values (methylation level) of the 7379 DMPs and the normalized expression of the previously identified 350 DERNAs [9]. Positive correlations are shown in green and negative correlations are shown in purple, with darker colour intensities indicating stronger correlation coefficients. (b) Overview of RNA groups compared for correlations between expression levels and methylation of identified DMPs, with the number of RNAs shown between brackets. (c) Comparison of correlation strength among RNA groups, expressed as the percentage of correlations with|rho| > 0.3. Differences between RNA groups were evaluated using a two‐proportion Z‐test. Asterisks (***) indicate *p* < 2.2 × 10^−16^. Abbreviations: DERNA, differentially expressed RNA; DMP, differentially methylated position.

**FIGURE 5:**
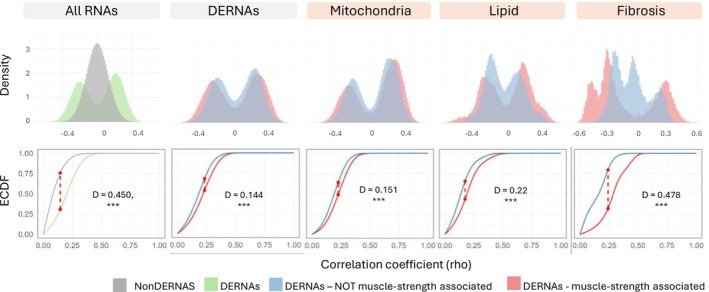
Histograms and statistical comparison of DMP correlations with different groups of RNA. Spearman's rank correlations between beta‐values of DMPs and normalized expression levels of DERNAs were computed for each RNA group. Histograms show the distribution of correlation coefficients within each group. Differences between distributions were assessed using the asymptotic two‐sample Kolmogorov–Smirnov (KS) test. The test statistic D, representing the maximum vertical distance between two distributions, is shown as a dashed line. Larger D values indicate larger differences between groups. All comparisons were significant (***indicates *p* < 2.2 × 10^−16^). Abbreviations: DERNA, differentially expressed RNA; DMP, differentially methylated position.

Correlations of the methylation levels of the 7379 DMPs with the expression levels of the 350 previously identified DERNAs in former ICU patients [9] are shown as a heatmap in Figure 4a. Overall, 18.1% of these DMP‐DERNA correlations had a|rho| > 0.3 (Figure 4b,c). In contrast, when correlating the same 7379 DMPs with the previously identified 16 335 non‐DERNAs [9], only 1.7% of the correlations showed|rho| > 0.3. This represents an absolute increase of 16.3% in the proportion of correlations with |rho| > 0.3 for DERNAs as compared with non‐DERNAs (*p* < 2.2 × 10^−16^ (lowest *p* value R can generate)). The proportion of correlations with |rho| > 0.3 was higher for DERNAs associated with muscle strength (24.4%) than for DERNAs not associated with muscle strength (12.5%, *p* < 2.2 × 10^−16^) (Figure 4b,c). This was also true when focusing on the previously identified persistently disturbed pathways [9]. Among DERNAs related to mitochondrial energy metabolism, 23.3% of those associated with muscle strength showed correlations with|rho| > 0.3 as compared with 10.9% of those not associated with muscle strength (*p* < 2.2 × 10^−16^). Similarly, for DERNAs related to lipid metabolism, these proportions were 15.9% as compared with 7.2% (*p* < 2.2 × 10^−16^). Fibrosis‐related DERNAs exhibited the most pronounced difference, with 44.3% for DERNAs associated with strength versus only 5.8% for DERNAs not associated with strength (*p* < 2.2 × 10^−16^). For each of the group comparisons, Kolmogorov–Smirnov tests showed significantly different distributions of the whole range of correlations (*p* < 2.2 × 10^−16^, Figure 5).

### 3.5 Risk Factors for Abnormal DNA Methylation in Muscle of Former ICU Patients

**FIGURE 6:**
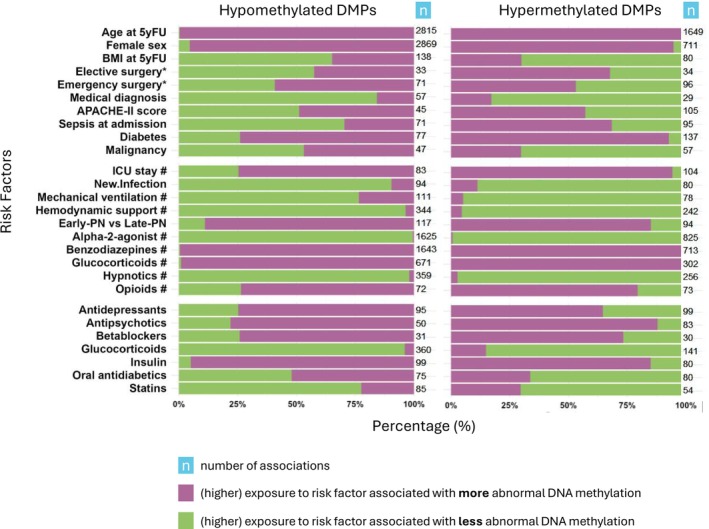
Risk factors for abnormal long‐term DNA methylation in muscle of former ICU patients. Associations between risk factors and abnormal DNA methylation are summarized, split up for hypomethylated and hypermethylated DMPs. The number of significant associations for each risk factor is indicated next to each bar. Negative associations with hypomethylated DMPs or positive associations with hypermethylated DMPs indicate that (higher) exposure to the risk factor associates with more abnormal and thus harmful DNA methylation, and vice versa. The x‐axis shows the percentage of associations with more abnormal (purple) or less abnormal (green) DNA methylation, calculated by dividing the number of harmful or protective associations by the total number of associations for each of the risk factors, separately for hypomethylated and hypermethylated DMPs. Abbreviations: DMP, differentially methylated position; ICU, intensive care unit; #, number of days.

Assessing whether certain factors may drive or protect against the observed abnormal long‐term DNA methylations in former ICU patients, we found 12 137 independent associations of such factors with hypomethylated positions and 6327 independent associations with hypermethylated positions (*p* < 0.05) (Figure 6), involving all DMPs. Risk factors associated with abnormal DNA methylation included non‐modifiable and potentially modifiable factors. Among the non‐modifiable factors, higher age was the most frequent, followed by (mostly female) sex. Concerning potentially modifiable ICU‐related factors, the highest proportions of associations with more abnormal DNA methylation were found for treatment with glucocorticoids and benzodiazepines (> 99%), followed by early‐PN (86.3%) and treatment with opioids (76.6%). With regard to medications taken at follow‐up, these proportions were highest for insulin (90.5%) and antipsychotics (84.2%).

## 4 Discussion

Muscle DNA of former ICU patients 5 years after critical illness showed differential methylation as compared with that of controls, with more hypomethylated than hypermethylated positions. A large proportion of the DMPs were intergenic. The DMPs associated with genes showed remarkable enrichment of genes involved in muscle contraction and signalling pathways. Genes harbouring differential methylation partially overlapped with the DERNAs previously identified in these former patients. Furthermore, correlation of the methylation level of the DMPs and the expression level of those DERNAs revealed a high proportion of biologically relevant correlations, particularly for DERNAs associated with muscle strength, also when focusing on DERNAs involved in specific persistently disturbed pathways (mitochondrial dysfunction, fibrosis and abnormal lipid metabolism). Several risk factors for the abnormal DNA methylation were identified, of which some are modifiable.

The epigenome is highly dynamic, constantly adapting to internal and external stressors [10, 24], including critical illness‐induced risk factors [13, 25, 26]. While some DNA methylation changes are transient, others may persist long after the initial insult, potentially contributing to long‐term sequelae [26, 27]. We identified 7379 DMPs in skeletal muscle DNA of former ICU patients 5 years after critical illness as compared with muscle DNA of controls, the majority being hypomethylated (63.6%). A predominance of DNA hypomethylation (75.6%) was previously shown to be present in skeletal muscle of adult patients during the critical illness phase, affecting many genes implicated in muscle homeostasis [13]. Likewise, abnormal DNA methylation in leukocytes or buccal mucosa of paediatric ICU patients during and long after ICU stay mostly involved hypomethylation [25, 26].

The observed abnormal DNA methylation patterns affected many genes. Functional annotation of hypomethylated genes revealed enrichment in pathways related to muscle contraction. Previous studies have shown that hypomethylation and active demethylation are linked to muscle contraction mechanisms in skeletal muscle and to muscle atrophy [13, 28, 29, 30]. Hypermethylated genes were enriched in pathways associated with vascular and epithelial branching morphogenesis and Ras signal transduction. Branched myofibers, which are more susceptible to damage, have been observed in dystrophy, trauma, aging and myotoxin exposure, suggested to be a consequence of myofiber remodelling during muscle regeneration [31]. Ras signal transduction is involved in muscle development [32]. Hence, the abnormal long‐term DNA methylation profile appears to affect several processes that are highly relevant for muscle function. This agrees with the abnormal DNA methylation observed in many processes that are highly relevant for muscle structure and function/weakness during ICU stay, which suggested plausibility of its involvement in the development of ICU‐acquired weakness [13]. Notably, while DNA methylation research has traditionally focused on gene promoters, we observed that most of the DMPs were located outside promoter regions, mainly in intergenic regions, but also in gene bodies and non–protein‐coding DNA. Non‐coding DNA substantially outnumbers coding DNA in the human genome. Altered methylation in non–protein‐coding or non‐coding DNA may exert its effect through regulatory mechanisms involving short and long non‐coding RNAs [33], as well as through local (cis) and distant (trans) mechanisms enabled by the 3D genome architecture (chromatin looping and inter‐chromosomal contacts) [19, 20].

DNA methylation partially shapes gene expression patterns, influencing cellular pathways and metabolic processes in skeletal muscle [12, 28, 30, 34]. We investigated whether long‐term abnormal DNA methylation associates with the previously observed long‐term abnormal RNA expression in muscle of former ICU patients [9]. Some genes were identified in the DNA methylome and transcriptome analyses, including several previously associated with reduced muscle strength. Several showed an inverse relation between promoter‐methylation and gene expression (e.g., down‐regulated ADGRF5 which compromises muscle stem cell pool maintenance [35] or up‐regulated NNMT, which negatively affects muscle strength [36]), consistent with canonical regulation [37]. Other genes did not strictly follow this rule of thumb, though they play important roles in muscle structure/function (e.g., SPTB and SYNPO2). This suggests that additional regulatory mechanisms beyond promoter methylation contribute to gene expression and functional impact in muscle. Other DMGs did not show abnormal expression and other DERNAs did not show abnormal DNA methylation. Several factors may contribute to this observation. On one hand, there may be a relation with the use of strict significance thresholds in both studies that therefore likely captured only a small fraction of the underlying complexity. On the other hand, this may reflect the dynamic and complex nature of DNA methylation and its effects. In this regard, overall, methylation effects on gene expression can be influenced by genomic location and sequence context, where methylation can also have long‐distance effects (even on other chromosomes, vide supra), as well as by interactions with other epigenetic modifications, contributing to regulatory disruptions through both direct and indirect pathways [19, 20, 38]. This is supported by our correlation analyses between altered DNA methylation and RNA expression. We observed a high percentage of biologically relevant correlations of DMP methylation levels with DERNA expression, also with DERNAs that did not harbour any DMP themselves. This percentage was much higher than for the correlations with non‐DERNAs, suggesting that DERNAs are more likely to be affected by methylation changes. Notably, the biologically relevant correlations were most pronounced for genes associated with reduced muscle strength [9], indicating that DNA methylation may show a more pronounced regulatory effect on genes contributing to long‐term muscle weakness. These included genes enriched in mitochondrial energy metabolism, fibrosis and lipid metabolism, as identified through previous transcriptome analyses [9]. DNA methylation has been implicated in the regulation of these pathways [39, 40, 41]. Our findings thus suggest that long‐term alterations in DNA methylation may relate to the documented long‐term disturbances in these pathways, associated with long‐term lower strength of former ICU patients [7, 8, 9].

The most pronounced risk factors for long‐term abnormalities in muscle DNA methylation were older age and female sex, which evidently are not modifiable. Also, several modifiable risk factors were found to be associated with abnormal DNA methylation. Regarding in‐ICU treatments, associations with more abnormal DNA methylation were most pronounced and consistent for benzodiazepines and glucocorticoids, followed by the early use of PN and opioids. Glucocorticoid treatment is a known risk factor for (ICU‐acquired) weakness [42, 43, 44] and, in the EPaNIC‐RCT, early‐PN was shown to aggravate the risk of ICU‐acquired weakness as compared with withholding PN for 1 week [14, 15]. Both treatments were recently also identified as pronounced risk factors for long‐term abnormal RNA expression in muscle after critical illness [9]. This suggests that their long‐lasting impact on the muscle may in part occur through long‐term changes in DNA methylation. The present data thus provide further rationale to limit exposure to glucocorticoids or reduce the dose and duration of treatment wherever possible, as well as further support against aggressive use of early full nutrition with early‐PN in the ICU. Benzodiazepine and opioid treatment in the ICU have been identified as risk factors for impaired long‐term physical function after critical illness [5], and particularly benzodiazepines have been associated with long‐term abnormal RNA expression [9], arguing for caution regarding their overzealous use as well.

The strengths of this study include the genome‐wide DNA methylation analysis of a very large number of CpG sites, spread over all chromosomes, allowing expansion of knowledge beyond gene functions that had not been implicated yet in long‐term muscle weakness. Next, stringent corrections for multiple comparisons ensured that the observed differences in DNA methylation were not findings by chance but rather remain a tip‐of‐the‐iceberg observation. Further strengths are the heterogeneity of the patient cohort and use of a control group; the study's embedding in a randomized controlled trial during which detailed patient information had been recorded at baseline, during ICU stay and at long‐term follow‐up; as well as the very long time window of follow‐up.

This study also has limitations, similar to our previous transcriptome study [9]. First, muscle biopsies could not be taken prior to ICU admission considering the largely unpredictable nature of critical illness. Hence, although patients who clearly showed impaired physical function already before ICU admission were excluded and alterations in DNA methylation can arise *de novo* during critical illness [25], it is possible that part of the abnormal DNA methylation in these ICU survivors may have been pre‐existing. Second, this is an observational study, with potential for unmeasured residual confounding. Hence, the correlations between abnormal DNA methylation and long‐term transcriptome changes, as well as the independent associations with risk factors, do not necessarily imply causal relationships. Even reverse causality remains theoretically possible, where part of the abnormal DNA methylation may result from weakness rather than causing it. One source of residual unmeasured confounding could be the lack of information on post‐ICU events. However, random post‐ICU exposures are expected to occur similarly in patients and controls, whereas non‐random exposures are expected to be intrinsic to the ICU population. Another source of confounding, specific to the risk factor analysis, could be the indication a drug was given for. However, the indication for glucocorticoid treatment in ICU was (at least partly) adjusted for by including sepsis, and allocation to early‐PN was random in the context of a randomized controlled trial. Third, although the patient sample size was large for this type of study, the relatively small control group may lack full representativeness, which may limit statistical sensitivity and underestimate differences between patients and controls. Fourth, no a priori power calculations were performed. Finally, we performed a very high number of statistical tests in different steps of this study. This carries inherent risk of type I error inflation with false positives, even with correction for multiple testing. Nevertheless, associations with clinical risk factors generate new hypotheses to be tested in future studies.

In conclusion, 5 years after ICU admission, muscle from former critically ill patients revealed abnormal DNA methylation as compared with control subjects, affecting pathways highly relevant for muscle function. Abnormal DNA methylation was associated with abnormal long‐term RNA expression, especially those RNAs associated with reduced long‐term muscle strength of the patients. Part of the abnormal DNA methylation related to the use of glucocorticoids and early‐PN, among other possibly avoidable risk factors. These findings could provide a biological basis for the long‐term persistence of weakness in ICU survivors and could open perspectives for prevention and possibly treatment of long‐term muscle weakness after critical illness through managing avoidable risk factors and modulating epigenetic regulation.

**Nomenclature**
DERNAs: Differentially expressed RNAs
DMGs: Differentially methylated genes
DMPs: Differentially methylated positions
ECDFs: Empirical cumulative distribution functions
FDR: False discovery rate
GOBP: Gene Ontology Biological Process
ICUAW: Intensive care unit‐acquired weakness
KEGG: Kyoto Encyclopedia of Genes and Genomes
Non‐DERNAs: Non‐differentially expressed RNAs
PN: Parenteral nutrition

## Funding

This work was supported by the Research Foundation—Flanders [Fonds Wetenschappelijk Onderzoek (FWO)], Belgium (research grant to IV (G039912), and research grant to IV and GVdB (G017325N)), by the Methusalem program of the Flemish government (through the KU Leuven to GVdB and IV, METH14/06) and by European Research Council Advanced Grants (AdvG‐2012‐321670, AdvG‐2017‐785809 and AdvG‐2023‐101133276) to GVdB. Part of the study was funded by the European Union. Views and opinions expressed are however those of the authors only and do not necessarily reflect those of the European Union or the European Research Council. Neither the European Union nor the granting authority can be held responsible for them.

## Ethics Statement

The Leuven University Hospital Ethics Committee approved the study protocol and informed consent forms (ML4190). The study has thus been performed in accordance with the ethical standards laid down in the 1964 Declaration of Helsinki and its later amendments. All participants gave their informed consent prior to inclusion in the study.

## Conflicts of Interest

The authors declare no conflicts of interest.

## Supporting information


**Figure S1:** Mean detection *p* values of each sample. Samples are clustered and coloured per chip. All samples have a mean detection *p* value < 0.05, which indicates satisfactory average signal detection above the background signal.
**Figure S2:** QC plot of DNA methylation data. The intensity of the methylated signal is plotted against the intensity of the unmethylated signal for each sample (depicted as circles). The dashed line illustrates the manufacturerprovided quality threshold. All samples exceeded this threshold and hence passed this overall quality control.
**Figure S3:** Density plot of DNA methylation data. This plot indicates the frequency of beta‐values over their full range (0 for no methylation to 1 for full methylation). Each line represents one sample, each colour represents one chip. Each sample shows a bi‐peak curve as expected, with large amounts of CpG sites that are either highly or hardly methylated.
**Figure S4:** Control strip plots of DNA methylation data. Control strip plots display the signal intensities of internal control probes that are designed to monitor various steps of the assay, including bisulphite conversion efficiency, non‐specific binding, hybridization, staining and extension. Consistent and expected patterns are observed which indicates good assay performance.
**Figure S5:** Evaluation of potential batch effects. The plot shows the visualization of the first two principal components of a principal component analysis (PCA), performed after stratified quantile normalization and adjusted for sex. Each dot represents one sample. Samples are grouped and coloured per chip. These three components together explain 5% of the observed variance in the dataset. There is no batch affect observed.

**Table S1:** Differentially methylated positions.
**Table S2:** Differentially methylated positions located within genes.
**Table S3:** Pathways enriched in DMGs in former ICU patients as compared with controls.
**Table S4:** Pathways enriched in DMP‐level in former ICU patients as compared with controls.
