# Supplementary material for: Association Between Abnormal DNA Methylation and Altered Transcriptome in Muscle Five Years After Critical Illness
Source: J Cachexia Sarcopenia Muscle. 2026 Jan 19;17(1):e70170. doi: 10.1002/jcsm.70170 (PMC12813554; doi:10.1002/jcsm.70170)
Supplement: Supplementary file 1 — Figure S1: Mean detection p values of each sample. Samples are clustered and coloured per chip. All samples have a mean detection p value < 0.05, which indicates satisfactory average signal detection above the background signal. Figure S2: QC plot of DNA methylation data. The intensity of the methylated signal is plotted against the intensity of the unmethylated signal for each sample (depicted as circles). The dashed line illustrates the manufacturerprovided quality threshold. All samples exceeded this threshold and hence passed this overall quality control. Figure S3: Density plot of DNA methylation data. This plot indicates the frequency of beta‐values over their full range (0 for no methylation to 1 for full methylation). Each line represents one sample, each colour represents one chip. Each sample shows a bi‐peak curve as expected, with large amounts of CpG sites that are either highly or hardly methylated. Figure S4: Control strip plots of DNA methylation data. Control strip plots display the signal intensities of internal control probes that are designed to monitor various steps of the assay, including bisulphite conversion efficiency, non‐specific binding, hybridization, staining and extension. Consistent and expected patterns are observed which indicates good assay performance. Figure S5: Evaluation of potential batch effects. The plot shows the visualization of the first two principal components of a principal component analysis (PCA), performed after stratified quantile normalization and adjusted for sex. Each dot represents one sample. Samples are grouped and coloured per chip. These three components together explain 5% of the observed variance in the dataset. There is no batch affect observed. [file JCSM-17-e70170-s001.pdf]

SUPPLEMENTARY APPENDIX

Association between abnormal DNA methylation and altered transcriptome in muscle five years after critical illness

Ceren **Uzun Ayar**, M.Sc.[1], Fabian **Güiza**, Ph.D.[1,2], Inge **Derese**, B.Sc.[1], Greet **Van den Berghe**, M.D.[1,2,#], Ilse **Vanhorebeek**, Ph.D.[1,#]

[1] Laboratory of Intensive Care Medicine, Department of Cellular and Molecular Medicine, KU Leuven, 3000 Leuven, Belgium; [2] Clinical Division of Intensive Care Medicine, University Hospitals Leuven, 3000 Leuven, Belgium; [#] Equally contributed

Corresponding author: Ilse Vanhorebeek, MEng, PhD Laboratory of Intensive Care Medicine, KU Leuven, Herestraat 49, B-3000 Leuven, Belgium; Tel +32 16 330532; Fax +32 16 344015; email ilse.vanhorebeek@kuleuven.be.

Supplementary Methods……………………………………………………………………………………3

Method S1. Differential RNA expression analysis……………………………………………………...……….4

Supplementary Figures ……………………………………………………………………………..……..5

Figure S1. Mean detection p-values of DNA methylation data per sample ………………………………………6
Figure S2. Overall quality plot of DNA methylation data……………………………………………………...7
Figure S3. Density plots of normalized DNA methylation data………………………………………………..8
Figure S4. Control strip plots of DNA methylation data…………………………………………………….....9
Figure S5. Principal component analysis of DNA methylation data after quantile normalization…...……...…..10

Supplementary Tables ………………………………………………………………...…………..…….11

Table S1. Differentially methylated positions……………….………………………………………...………12
Table S2. Differentially methylated positions located within genes…..……………………………...…...….12
Table S3. Pathway enrichments of differentially methylated genes…….……………………………………..12
Table S4. Pathway enrichments of differentially methylated positions ..………………………..……………..12

References ……………………………………………………………………………………………….13

SUPPLEMENTARY METHODS

Method S1. Differential RNA Expression

The differential RNA expression analysis used in the present study has been described previously [1]. RNA was extracted with use of an in-house protocol, consisting of tissue lysis with QIAzol Lysis Reagent (Qiagen, Venlo, The Netherlands) and isopropanol precipitation. For one sample, yield was insufficient. Further sample preparation and total RNA sequencing were performed by the UZ/KU Leuven Genomics Core Facility. Sample purification was performed with Qiagen's MinElute purification kit. Extracted RNA was checked for quality, libraries were prepared (Bioké NEBNext kit for Illumina) and unique identifiers were added. This process failed for two samples. High-throughput paired-end RNA sequencing was performed with the Illumina Novaseq platform. Next, data trimming was conducted with Trimmomatic [2] and the quality was assessed with FastQC [3] and SortMeRNA [4]. Reads were mapped to the human genome (GRCh38) with HISAT2 [5], yielding read counts for each transcript. RNAs with read counts above 10 in fewer than 30 samples (the smallest sample group) were excluded, as recommended [6]. Two samples with low total read counts were excluded for further analyses. We obtained an average of approximately 27.8 clean million reads per sample, aligned to the human reference genome with a total mapping rate of 93.6-98.5%. After excluding RNAs that did not have at least 10 copies in at least 30 samples [6], we retained 16,685 RNAs for differential expression analysis. Differential expression analysis was performed with the DEseq2 package in R [6]. For quality control, hierarchical clustering to the sample-to-sample distance matrix was applied, accounting for covariates (age, sex, BMI), and the first 30 Principal Components (PCs) were investigated. This revealed one outlier sample exhibiting high variability that was therefore excluded. The input matrix for the differential expression analysis contained unnormalized read counts for each retained RNA in each retained sample. RNAs differentially expressed between former ICU patients and controls were identified while adjusting for demographics (sex, age and BMI). The continuous covariates (age and BMI) were centered following guidelines [6]. DESeq2 internally normalized the input matrix for sequencing depth and RNA composition. The Benjamini-Hochberg procedure was applied for multiple testing correction, defining significant differential expression with a false-discovery-rate (FDR) lower than 0.05.

SUPPLEMENTARY FIGURES

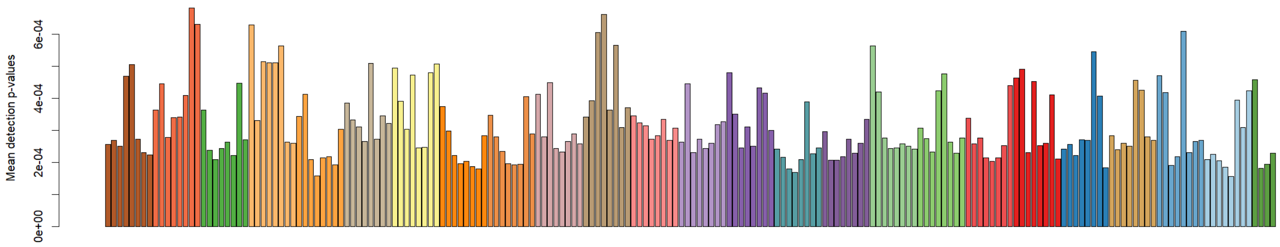

Figure S1. Mean detection *P*-values of each sample. Samples are clustered and colored per chip. All samples have a mean detection *P*-value <0.05, which indicates satisfactory average signal detection above the background signal.

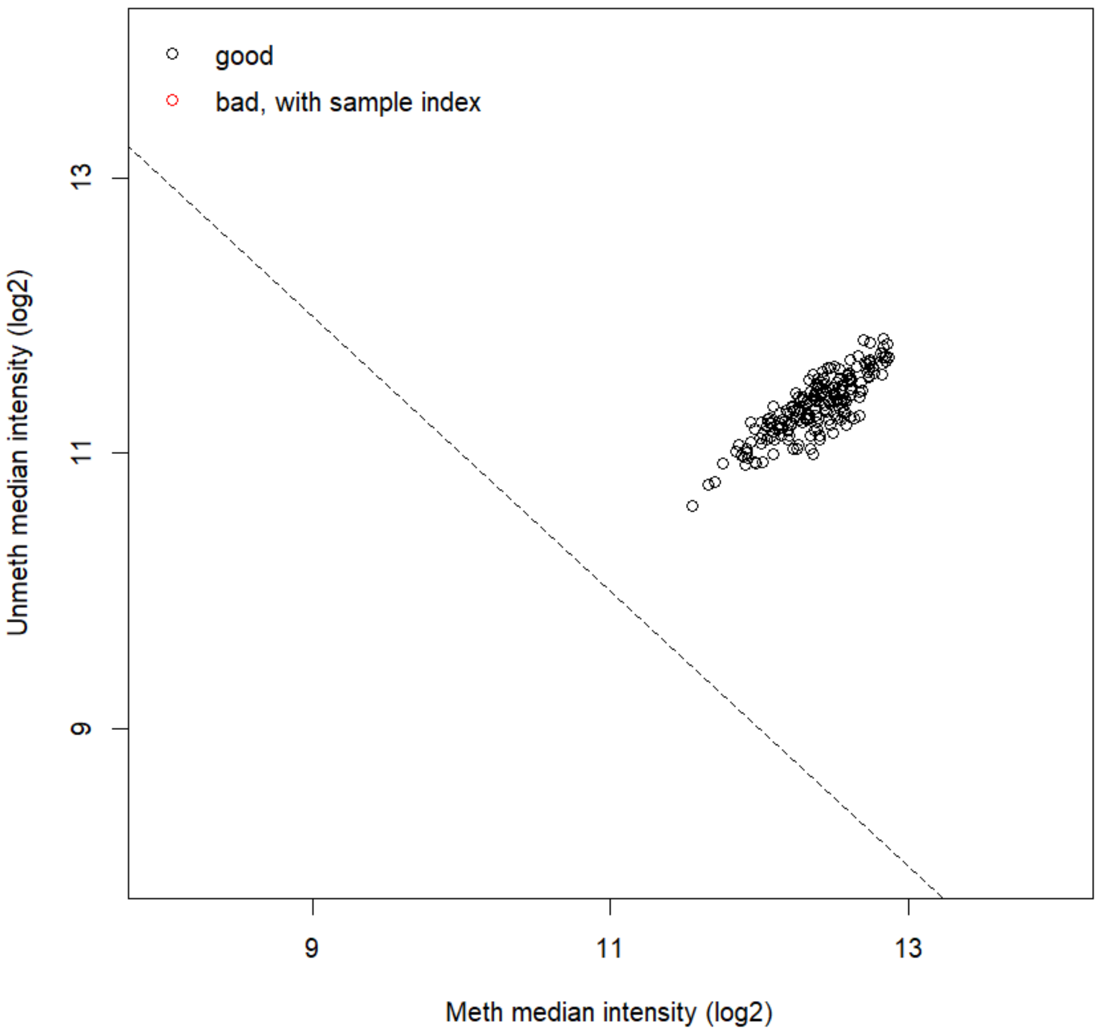

Figure S2. QC plot of DNA methylation data. The intensity of the methylated signal is plotted against the intensity of the unmethylated signal for each sample (depicted as circles). The dashed line illustrates the manufacturer-provided quality threshold. All samples exceeded this threshold and hence passed this overall quality control.

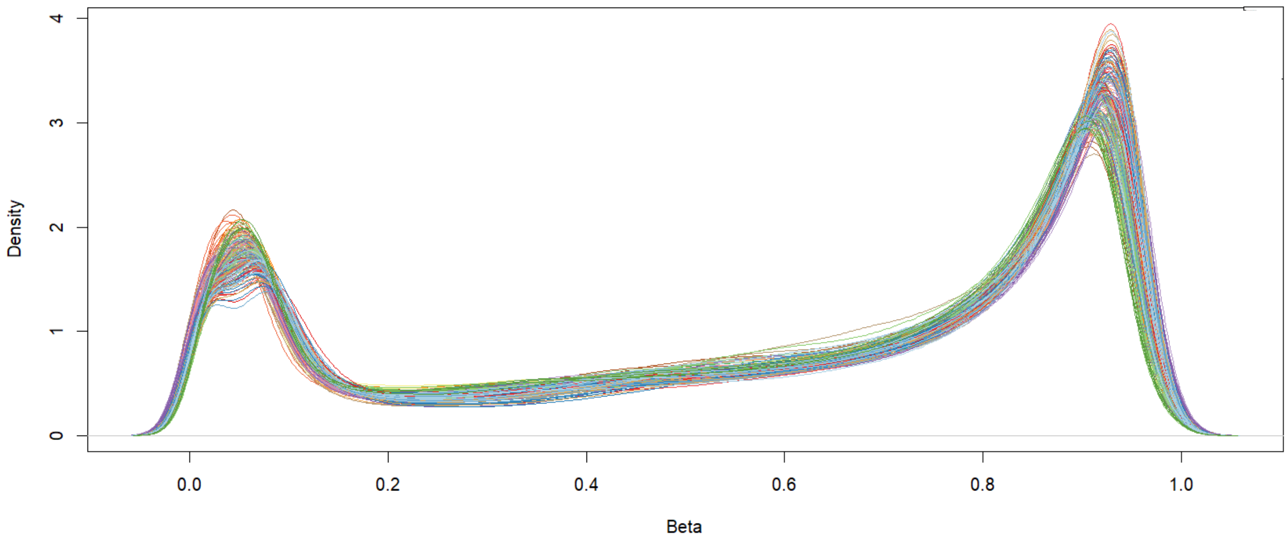

Figure S3. Density plot of DNA methylation data. This plot indicates the frequency of beta-values over their full range (0 for no methylation to 1 for full methylation). Each line represents one sample, each color represents one chip. Each sample shows a bi-peak curve as expected, with large amounts of CpG sites that are either highly or hardly methylated.

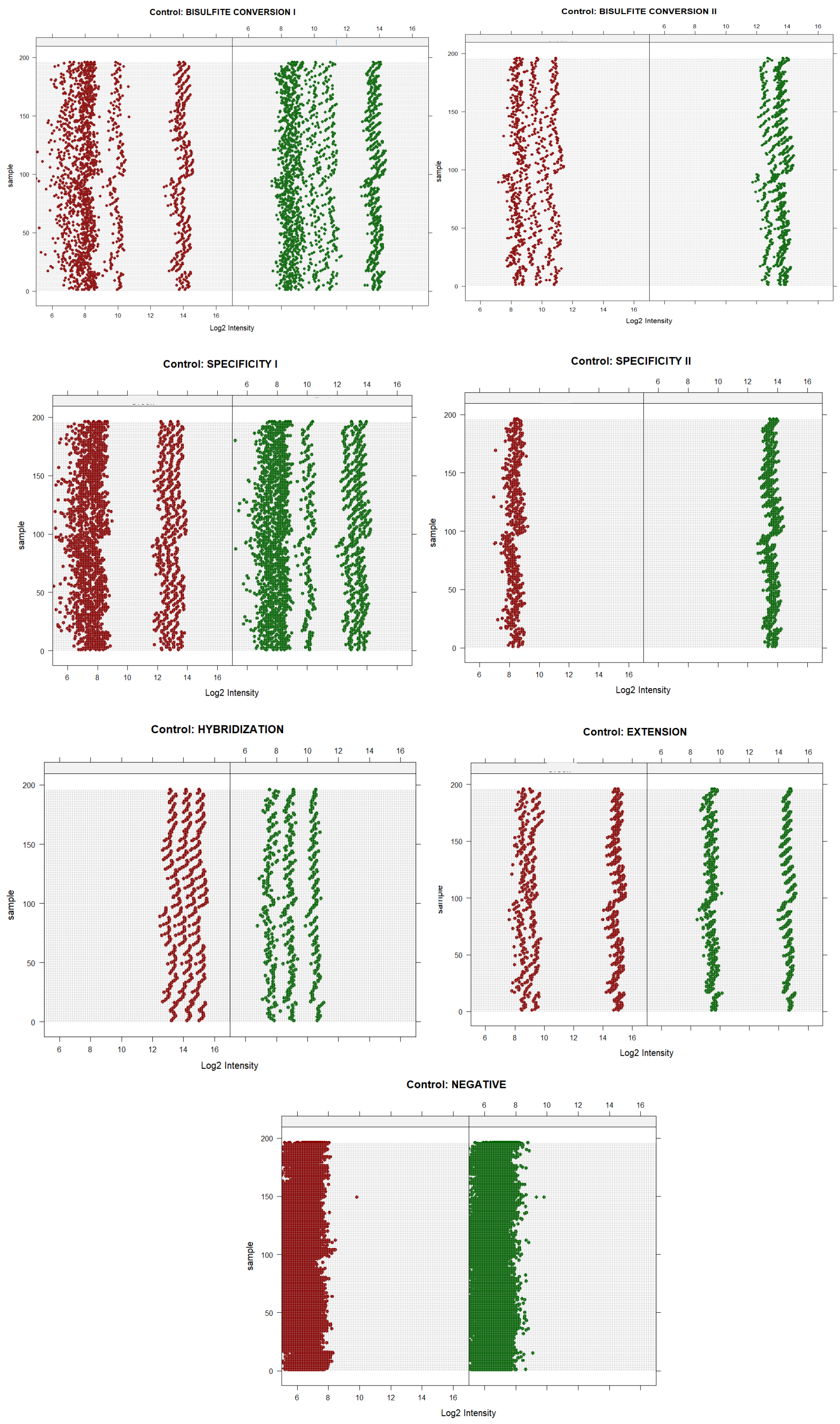

Figure S4. Control strip plots of DNA methylation data. Control strip plots display the signal intensities of internal control probes that are designed to monitor various steps of the assay, including bisulfite conversion efficiency, non-specific binding, hybridization, staining and extension. Consistent and expected patterns are observed which indicates good assay performance.

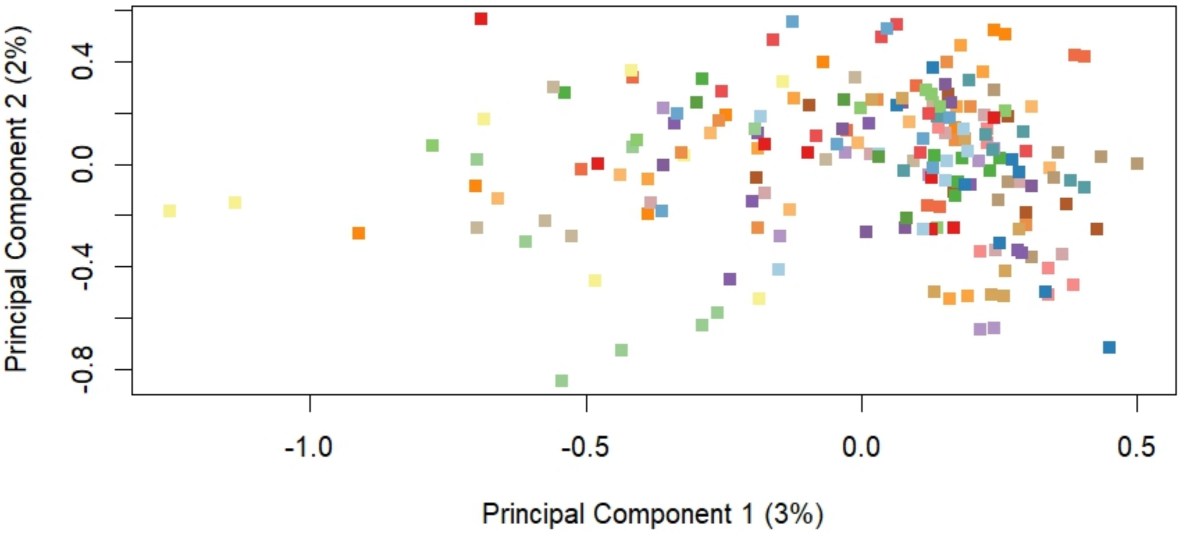

Figure S5. Evaluation of potential batch effects. The plot shows the visualization of the first two principal components of a principal component analysis (PCA), performed after stratified quantile normalization and adjusted for sex. Each dot represents one sample. Samples are grouped and colored per chip. These three components together explain 5 % of the observed variance in the dataset. There is no batch affect observed.

SUPPLEMENTARY TABLES

Table S1. Differentially methylated positions

Table S2. Differentially methylated positions located within genes

Table S3. Pathway enrichments of differentially methylated genes

Table S4. Pathway enrichments of differentially methylated positions

Tables are provided as a supplementary excel file.

References

1. Uzun Ayar C, Güiza, F., Derese, I. et al. Altered muscle transcriptome as molecular basis of long-term muscle weakness in survivors from critical illness. Intensive Care Med. 2025;doi:10.1007/s00134-025-07949-3
2. Bolger AM, Lohse M, Usadel B. Trimmomatic: a flexible trimmer for Illumina sequence data. Bioinformatics. 2014;30:2114-20. doi:10.1093/bioinformatics/btu170
3. Andrews S. FastQC: A Quality Control Tool for High Throughput Sequence Data. 2010;
4. Kopylova E, Noé L, Touzet H. SortMeRNA: fast and accurate filtering of ribosomal RNAs in metatranscriptomic data. Bioinformatics. 2012;28:3211-7. doi:10.1093/bioinformatics/bts611
5. Kim D, Paggi JM, Park C, Bennett C, Salzberg SL. Graph-based genome alignment and genotyping with HISAT2 and HISAT-genotype. Nat Biotechnol. 2019;37:907. doi:10.1038/s41587-019-0201-4
6. Love MI, Huber W, Anders S. Moderated estimation of fold change and dispersion for RNA-seq data with DESeq2. Genome Biol. 2014;15:550 doi:10.1186/s13059-014-0550-8
